# Perceived Unfair Police Treatment and Recent Substance Use Among Male Youth Residing in Pittsburgh: Cross-Sectional Findings from a Randomized Control Trial

**DOI:** 10.3390/ijerph23070852

**Published:** 2026-06-30

**Authors:** Lynissa R. Stokes, Alison J. Culyba, Amber L. Hill, Elizabeth Miller

**Affiliations:** 1 Department of Pediatrics, University of Pittsburgh School of Medicine, Pittsburgh, PA 15224, USA; alison.culyba@chp.edu (A.J.C.); alh198@pitt.edu (A.L.H.); elizabeth.miller@pitt.edu (E.M.); 2 Division of Adolescent and Young Adult Medicine, UPMC Children’s Hospital of Pittsburgh, Pittsburgh, PA 15213, USA; 3 Latouche Pediatrics, Anchorage, AK 99508, USA; 4 Integrated Center for Women’s Health, National Center for Child Health and Development, Tokyo 157-8535, Japan

**Keywords:** discrimination, minority youth, male youth, tobacco use, alcohol use, marijuana use, police

## Abstract

**Highlights:**

**Public health relevance—How does this work relate to a public health issue?**

- Substance use among minority youth remains a critical public health issue.
- This study examined whether race-based discrimination, a common stressor in the lives of minority youth, is associated with past 30-day tobacco, alcohol, and marijuana use.

**Public health significance—Why is this work of significance to public health?**

- While race-based discrimination in the lives of minority youth is well-documented, this study identified unfair police treatment as a specific stressor associated with significantly higher odds of tobacco use and marijuana use.
- Study findings highlight the need to evaluate specific types of race-based discrimination, rather than relying solely on aggregate scores, when assessing associations with substance use among minority youth.

**Public health implications—What are the key implications or messages for practitioners, policy makers and/or researchers in public health?**

- Public health researchers are encouraged to move beyond using composite or aggregate discrimination scores to include item-specific measurement to better understand how different types of discriminatory events, such as law enforcement contact, relate to substance use and other problem behaviors among minority youth.
- Addressing the specific impact of unfair police treatment, rather than general race-based discrimination alone, is essential for developing targeted public health policies and interventions to improve the well-being of minority youth.

**Abstract:**

This study examined associations between race-based discrimination and substance use among a predominantly minority sample of male youth, ages 13–19, residing in under-resourced neighborhoods in Pittsburgh, Pennsylvania. Youth completed in-person, anonymous electronic surveys about their lifetime experiences with race-based discrimination and past 30-day tobacco, alcohol, and marijuana use. Logistic regression models examined associations between race-based discrimination, measured as a sum of each discriminatory experience endorsed and by each individual discriminatory experience, and past 30-day substance use. Models controlled for age, sexual orientation, school suspension history, parental education, and intervention status. Although most youth (85.6%) reported experiencing one or more forms of race-based discrimination in their lifetime, total discrimination scores were not significantly associated with past 30-day substance use. However, item-specific analyses revealed that unfair police treatment was significantly associated with greater odds of tobacco use (AOR = 2.18; 95% CI 1.39, 3.41) and marijuana use (AOR = 2.26; 95% CI 1.51, 3.39). Study findings highlight the importance of item-specific measurement since types of race-based discrimination have different consequences for youth (e.g., detention from an unfair teacher, police arrest). Study results suggest the need for healing-centered strategies and policy reforms to address the negative impact of policing on minority youth’s well-being.

## 1. Introduction

Discrimination is a common stressor in the lives of minority youth, with some evidence indicating that they experience race-based micro- and macroaggressions daily [1,2]. Microaggressions are defined as subtle derogatory slights, such as a teacher recommending less rigorous classes for students of color or someone questioning a person’s country of origin (e.g., “Where are you really from?”) [3]. Macroaggressions, conversely, refer to overt acts of race-based discrimination, such as the uneven use of school disciplinary policies that often disproportionately affect students of color [4,5].

Exposure to race-based discrimination has been linked to impaired physical and mental health [1,2,6,7,8,9], negative interpersonal interactions [9,10,11,12,13], and risky behaviors [9,14] among diverse populations of adolescents. Although associations between race-based discrimination and adolescent substance use are well-established [15,16,17,18,19,20], some gaps in the literature remain. Specifically, not all discriminatory experiences occur at the same frequency [1,8,20,21] or have the same impact on those who experience them [6,8,15,20,22]. Studies about race-based discrimination, however, tend to use total or average discrimination scores or categorize discrimination as dichotomous (e.g., none vs. any) in their analyses [7,8,15,18].

Assessing the impact of race-based discrimination on youth based on the sum, average, or the presence or absence of discriminatory experiences, regardless of type, could potentially obscure some of the ways in which specific types of discrimination relate to adolescent well-being and functioning. For example, unfair treatment by teachers has been shown to undermine school engagement and performance in minority youth [23,24,25]. Benner and Graham [26] found that discrimination from peers was related to psychological maladjustment, while societal discrimination (e.g., being hassled by police, unfair treatment by a store clerk) was associated with heightened racial awareness in a sample of ethnically diverse adolescents. Witnessing the mistreatment of family members due to their race has been linked to hypervigilance and anxiety among youth, even when they were not the direct target of the encounter [27].

Among the myriad types of race-based discrimination youth may encounter, those involving law enforcement can have traumatic, sometimes even fatal consequences [28,29]. Badolato and colleagues [28], for example, found that over a 16-year period, over one hundred youth, between the ages of 12 and 17, died as a result of a police interaction. Nonfatal interactions with police can also have negative implications for youth. For instance, Jackson and colleagues [29] found that youth who were frequently stopped by police were more likely to experience emotional distress and post-traumatic stress symptoms. Wiehe and colleagues [20] found that unfair police treatment was associated with increased odds of smoking among Black and Latino adolescent males. Taken together, these studies illustrate that different discriminatory experiences can have a wide range of negative implications for youth well-being. Therefore, there is a continuing need for research that disaggregates these experiences rather than only using average or total discrimination scores.

The purpose of this study was to examine self-reported race-based discrimination and its association with past 30-day substance use among a predominantly minority sample of male youth. Specifically, we examined whether discrimination in general and different categories of discriminatory experiences were associated with past 30-day tobacco, alcohol, and marijuana use among a sample of Pittsburgh male youth between the ages of 13 and 19. Our study builds on the work of Wiehe and colleagues [20] by using a discrimination measure developed specifically for youth that includes items that assess both micro- and macroaggressions due to race [30] and broadens the scope of substance use beyond smoking to include alcohol and marijuana use.

## 2. Methods

### 2.1. Participants

Male youth were recruited through community agencies in Pittsburgh, Pennsylvania, to participate in either Manhood 2.0 or a job readiness program. Manhood 2.0 is a community-based gender-transformative program containing a curriculum that focuses on three main topics: gender norms, violence and its impact on communities, and sexual and reproductive health. The job readiness program, which served as the control condition, reviewed resumé creation, developing interview skills, and other topics related to successful job-seeking strategies. To be eligible for the randomized control trial, youth needed to self-identify as male, be between 13 and 19 years old, and reside in a socially disadvantaged neighborhood in Pittsburgh, Pennsylvania. Recruitment was done with assistance from community leaders and staff at youth-serving agencies, with programming occurring in places of worship, libraries, community centers, juvenile justice organizations, and a minority-serving organization located in socially disadvantaged neighborhoods [31].

### 2.2. Data Collection

Research assistants obtained verbal assent before the youth completed anonymous electronic surveys. Youth received compensation for completing the end-of-program surveys, which was the only time questions about substance use and race-based discrimination were asked. The University of Pittsburgh’s Institutional Review Board (IRB) approved the study protocol with a waiver of parental permission. The IRB granted the waiver of parental consent because the parent study [31] was deemed to be of minimal risk and included questions about sensitive topics (e.g., substance use, discrimination, sexual behavior) such that requiring parental involvement could introduce selection bias, compromise participant privacy, or deter youth from participating in research addressing these topics. Due to the sensitive nature of the topics included in the surveys, a formal distress protocol was created to protect participants’ well-being.

### 2.3. Measures

Youth provided information about their sociodemographic characteristics, educational status, year in school (if applicable), caregiver’s highest level of education (which served as a proxy for socioeconomic status), and school suspension history.

We assessed race-based discrimination by asking whether youth had ever experienced any of ten situations because of their skin color, language or accent, or culture in their lifetime [30]. The measure used, Perceptions of Racism in Children and Youth (PRaCY), was validated in a separate study of minority male and female youth residing in Pittsburgh [10]. We calculated a total discrimination score (0–10) based on the sum of “yes” responses and also included the individual discriminatory experiences in analyses.

Youth answered questions about the frequency of past 30-day tobacco, alcohol, and marijuana use. Tobacco, alcohol, and marijuana use variables were dichotomized (0 = no use, 1 = any use) to evaluate the occurrence rather than frequency of use. This was done because preliminary analyses indicated that past 30-day substance use data were highly skewed with low overall base rates.

### 2.4. Statistical Analysis

Descriptive statistics were used to summarize sociodemographic characteristics, discriminatory experiences, and substance use. Covariates included in the analyses were participant age, sexual orientation, history of school suspension (yes/no), and parent/caregiver’s highest level of education, each of which has been associated with discrimination exposure or substance use [12,32,33,34,35,36] in relevant previous studies with adolescent populations. We also controlled for whether participants were in the Manhood 2.0 group (*n* = 306) or the job readiness program control group (*n* = 242).

Logistic regression models were used to examine associations between discrimination (total score and each form of discrimination) and past 30-day tobacco, alcohol, and marijuana use. To account for the risk of Type I error associated with conducting multiple comparisons across discrimination items and substance use types, we applied a conservative significance threshold of *p* < 0.002 (0.05 ÷ 33 comparisons). Analyses were conducted using SPSS Version 31 (SPSS Inc., Chicago, IL, USA).

## 3. Results

A total of 577 youth completed the end-of-program survey. However, only 548 youth were included in analyses after 29 participants were excluded due to missing data for the primary variables of interest. Specifically, participants were removed if they: (a) did not provide responses for any of the discrimination or substance use questions (*n* = 14), (b) did not answer any of the discrimination questions (*n* = 7), (c) did not answer any of the substance use questions (*n* = 5), or (d) answered only one of the ten discrimination questions (*n* = 3). For all other variables included in analyses (e.g., race, ethnicity, sexual orientation), skipped responses were coded as “no response provided.”

As shown in Table 1, the average participant age was 15.5 years old (*SD* = 1.6). Most of the participants identified as Black/African American (71.9%), were born in the United States (90.7%), and identified as heterosexual (81.4%). Most of the participants were in school at the time of data collection (87.8%) and had a history of being suspended from school (70.8%). The most prevalent level of educational attainment for participants’ parent/caregiver was less than high school (42.5%), followed by college degree or higher (25.9%), completed high school (17.5%), and some college (8.4%).

Race-based discrimination was reported by most of the youth (85.6%). The largest proportion of youth reported being accused of something they didn’t do in school (66.2%). Vicarious discrimination was the least common form of race-based discrimination reported (27.2%). Table 2 summarizes each form of discrimination reported by youth. Also shown in Table 2, a total of 24.3% of youth reported past 30-day tobacco use, 22.8% reported past 30-day alcohol use, and 33.6% of youth reported past 30-day marijuana use. In the past 30 days, 58.8% of participants reported no use of any substances, 17.9% reported using only one substance, and 23.4% reported using two or more substances.

Total discrimination was unrelated to past 30-day tobacco, alcohol, or marijuana use (Table 3). Of the ten items included in the PRaCY scale, unfair police treatment was associated with greater odds of tobacco (AOR = 2.18; 95% CI: 1.39–3.41) and marijuana use (AOR = 2.26; 95% CI: 1.51–3.39). The association between unfair police treatment and alcohol use did approach significance (AOR = 2.05; 95% CI: 1.29–3.26), based on the adjusted significance level *p* < 0.002.

## 4. Discussion

Our study expands the research on associations between race-based discrimination and substance use among male youth by examining both overall discrimination and individual discriminatory experiences. We did not find any significant associations between overall discrimination and past 30-day substance use among the youth in our study. However, associations were found between unfair police treatment, which was reported by approximately 40% of the youth in our study, and past 30-day tobacco use and marijuana use. The association between unfair police treatment and past 30-day alcohol use approached significance.

There is ample evidence that youth interactions with police can have a wide range of negative consequences [28,29,37,38,39]. For example, a recent systematic review summarized significant associations between police exposure (defined as police contact or perceived police discrimination) and adverse mental health, sexual risk behaviors, and substance use among Black youth [37]. In a separate systematic review, Oppenheim and colleagues found that police violence, whether direct, vicarious, or anticipated, was associated with traumatic stress in youth [39]. Our study can be added to this body of work, particularly since unfair police treatment was the only form of race-based discrimination significantly associated with past 30-day tobacco use and marijuana use.

Furthermore, our study highlights the importance of using both global and item-specific measurement approaches to assess associations between race-based discrimination and youth substance use. Had we limited our analyses to overall discrimination, associations between unfair police treatment and substance use would not have been revealed. In addition, our findings align with those of Wiehe and colleagues [20], who also found that perceived discrimination during police interactions was associated with increased odds of smoking in their sample of Black and Latino adolescent males. Both our study and theirs used a cross-sectional design. Therefore, further exploration of associations between race-based discrimination (globally and by type of discrimination) and substance use, utilizing a longitudinal study design, is warranted.

Some study limitations should be noted. First, most of the youth self-identified as Black/African American and heterosexual. Therefore, it is unclear whether similar associations would be found between race-based discrimination and substance use in other groups of racial and ethnic minority youth. Second, youth were not asked whether they identified as transgender or non-binary. In addition, the sample of sexual minority-identifying youth was too small for between-group analyses.

Although race-based discrimination is a common experience for many minority youth [1,2], the nature and frequency of discrimination may differ based on intersecting social identities (e.g., being Black and transgender). This observation is supported by a cross-sectional study of over 3000 high school students, which found that the highest rates of social identity-based victimization were among youth who reported multiple marginalized social identities (e.g., racial/ethnic minority status and LGBTQ status) [12]. Neither that study nor our study stratified results by multiple social identities. Therefore, future studies, sampling a larger and more diverse group of youth, could provide opportunities to analyze data by intersecting identities to explore how generalizable our findings are among youth with multiple social identities exposed to race-based discrimination and other forms of social identity-based discrimination.

Due to the cross-sectional nature of our study, it is unclear whether past 30-day substance use led to interactions with police that were perceived as unfair by male youth or if substance use followed unfair police interactions as a method of coping. Support for the former possibility comes from a study that found that African American youth were more likely to be arrested despite lower rates of drug use compared to their white peers [40]. Support for the latter comes from a study by Gibbons and colleagues [41] that found that African American youth’s experiences with race-based discrimination were related to later substance use.

Associations between race-based discrimination and substance use could also be bidirectional. Youth who use substances may be more likely to have contact with law enforcement, and these encounters, particularly if they involve being searched or aggressive questioning, may be perceived by the youth as unfair or racially biased. Future longitudinal research is needed to determine if discriminatory policing acts as a traumatic stressor that then leads to substance use, if substance use among minority youth increases the frequency of negative police interactions, or if the relationship is bidirectional.

Lastly, the youth recruited for the study resided in under-resourced neighborhoods in Pittsburgh, Pennsylvania. Therefore, it is unclear whether exposure to race-based discrimination and rates of substance use found among our study participants are comparable to those of youth residing in other Pittsburgh neighborhoods. This limits the generalizability of our findings, particularly as there is some evidence that minority youth experience more discrimination in affluent neighborhoods [42]. Future studies should consider sampling youth from neighborhoods of varying levels of affluence to examine whether associations between race-based discrimination and substance use vary by location.

Our study found that race-based discrimination in the form of unfair police treatment is not an uncommon experience for minority youth and is associated with recent tobacco and marijuana use. These findings, along with those previously cited [28,29,37,38,39,40,41], support the ongoing need for strategies that go beyond helping youth prepare for or manage police interactions [43] to shifting to fundamentally restructuring how law enforcement interacts with minority youth. Such strategies should include evaluating whether law enforcement should be present in schools, where minority youth often face harsher disciplinary practices [4,5]. For example, a systematic review of school-based law enforcement (SBLE) found that, when compared to schools without SBLE, their presence was associated with higher rates of exclusionary discipline (e.g., suspensions, expulsions) and was an ineffective method for improving school safety [44].

Given the prevalence of unfair policing and its negative impact on minority youth, strategies to mitigate associations between this form of race-based discrimination and substance use are needed. Gibbons and colleagues [41] found that the relationship between race-based discrimination and later substance use was mediated by anger, but also that supportive parenting moderated associations between race-based discrimination and later substance use. The measure to assess race-based discrimination did not, however, include unfair police treatment. Oppenheim and colleagues [39] recommended that mental health professionals be trained to support youth exposed to police violence. This recommendation, along with the findings from Gibbons and colleagues [41], suggest that such training should include ways to effectively manage anger.

## 5. Conclusions

Substance use may represent one method of coping for minority youth who experience race-based discrimination [41,45], which can include unfair police treatment. Youth-serving medical and mental health professionals should consider race-based discrimination as a potential influence on health-compromising behaviors among the minority youth they treat. Anti-racism educational tools and resources for youth-serving professionals should include information about navigating the trauma associated with police interactions. Finally, strategies designed to combat race-based discrimination and its impact on minority youth, such as institutional (e.g., schools, law enforcement) policy reforms and community-led initiatives, are needed.

## Figures and Tables

**Table 1:** Sociodemographic characteristics (*N* = 548).

**Age (in Years)**	*M* = 15.5
	*SD* = 1.6
	% (n)
**Race/Ethnicity**	
Black/African American	71.9 (394)
White	3.1 (17)
Hispanic	5.7 (31)
Multiracial	10.0 (55)
Other	5.5 (30)
No Response	3.8 (21)
**Born in the U.S.**	
Yes	90.7 (497)
No	3.8 (21)
No Response	5.5 (30)
**Sexual Orientation**	
Heterosexual	81.4 (446)
Sexual Minority	9.1 (50)
No Response	9.5 (52)
**Education Status**	
Currently in School	87.8 (481)
Not in School	7.1 (39)
No Response	5.1 (28)
**School Suspension History**	
Yes	70.8 (388)
No	28.5 (156)
No Response	0.7 (4)
**Parental Education**	
Did Not Complete High School	42.5 (233)
Completed High School	17.5 (96)
Some College	8.4 (46)
College Degree or Higher	25.9 (142)
No Response	5.7 (31)

Notes. All participants who selected Hispanic also selected at least one race and were only included in the total for Hispanic participants. The Multiracial category includes youth who selected more than one race (and did not select Hispanic) or who selected Multiracial (and did not select Hispanic). The Other category includes youth who self-identified as Asian, Native Hawaiian or Pacific Islander, Native American or Alaskan Native, or who selected Other (and did not select Hispanic). Response options for sexual orientation were heterosexual, mostly heterosexual, bisexual, mostly homosexual, and homosexual. Due to small cell sizes across the individual non-exclusively heterosexual response categories, responses were re-coded for analyses into a dichotomous variable (heterosexual and sexual minority) for participants who selected an option other than “heterosexual”.

**Table 2:** Discrimination experiences and past 30-day substance use.

**Lifetime Discrimination Experiences**	% (n)
Overall discrimination (% who reported one or more types of discrimination)	85.6 (469)
Watched by security guards or store clerks	39.8 (218)
Treated unfairly by a police officer	37.8 (207)
Accused of something you didn’t do at school	66.2 (363)
Treated unfairly by a teacher	51.8 (284)
Felt that someone was afraid of you	52.2 (286)
Called an insulting name	52.4 (287)
Someone made a bad remark about your race, ethnicity, or language	41.6 (228)
Someone was rude to you	55.3 (303)
People assume you’re not smart or intelligent	48.7 (267)
Saw your parents or family member treated unfairly	27.2 (149)
**Past 30-Day Substance Use by Substance**	% (n)
Tobacco	24.3 (133)
Alcohol	22.8 (125)
Marijuana	33.6 (184)

Note. Percent of youth who responded “yes”.

**Table 3:** Associations between lifetime race-based discrimination and past 30-day substance use.

Lifetime Discrimination	Past 30-Day Substance Use		
	Tobacco Use	Alcohol Use	Marijuana Use
	AOR (95% CI)	AOR (95% CI)	AOR (95% CI)
Total discrimination	1.06 [0.98, 1.14]	1.04 [0.96, 1.13]	1.04 [0.98, 1.12]
Watched closely or followed around by security guards or store clerks	1.01 [0.65, 1.59]	1.62 [1.02, 2.56] ^b^	1.77 [1.18, 2.64] ^b^
Were treated unfairly by a police officer	**2.18** **[1.39, 3.41]**	2.05 [1.29, 3.26] ^a^	**2.26** **[1.51, 3.39]**
Accused of something you didn’t do at school	0.86 [0.53, 1.39]	0.79 [0.49, 1.29]	0.81 [0.52, 1.24]
Treated badly or unfairly by a teacher	1.36 [0.87, 2.12]	1.09 [0.69, 1.71]	1.17 [0.79, 1.74]
You had the feeling that someone was afraid of you	1.66 [1.06, 2.61] ^b^	1.52 [0.96, 2.41]	1.58 [1.06, 2.35] ^b^
Someone called you an insulting name	1.17 [0.75, 1.83]	1.10 [0.70, 1.74]	0.92 [0.62, 1.37]
Someone made an insulting remark based on race, ethnicity, or language	1.11 [0.71, 1.72]	0.97 [0.61, 1.53]	0.91 [0.61, 1.35]
Someone was rude	0.91 [0.58, 1.43]	0.76 [0.48, 1.21]	0.85 [0.56, 1.28]
People assume you’re not smart or intelligent	0.93 [0.60, 1.44]	0.78 [0.50, 1.23]	0.87 [0.59, 1.30]
Saw parent or caregiver unfairly treated	1.43 [0.89, 2.29]	1.87 [1.16, 3.02] ^b^	1.28 [0.83, 1.97]

Notes. AOR = adjusted odds ratio; CI = confidence interval. Odds ratios adjusted for age, sexual orientation, parent/caregiver highest level of education, school suspension history, and intervention assignment. Total discrimination based on the sum of all “yes” responses for each discrimination item. To protect against Type I error, we applied a Bonferroni *p*-value correction to the test for associations between discrimination (total and by item) and substance use. A *p*-value < 0.002 was considered significant (0.05/33 tests = 0.002). Numbers in bold indicate significant findings at that adjusted *p*-value. One item approached significance (*p*-value = 0.002) and is marked with superscript ^a^. Items with a *p*-value between 0.005 and 0.05 are marked with subscript ^b^.

## Data Availability

The data that support the findings of this study will be made available upon reasonable request. Due to the sensitive nature of the information gathered for the study and the fact that most of the study participants were minors, restrictions apply to the availability of these data to ensure participant privacy and compliance with ethical guidelines. In accordance with the IRB-approved protocol, data sharing will be subject to a formal data use agreement and must align with the information outlined in the participant assent document used in the study.

## Abbreviations

The following abbreviations are used in this manuscript:

AOR	Adjusted odds ratio
PRaCY	Perceptions of Racism in Children and Youth
LGBTQ	Lesbian, gay, bisexual, transgender, and queer or questioning
SBLE	School-based law enforcement
